# Soy Protein-Based Infant Formulas with Supplemental Fructooligosaccharides: Gastrointestinal Tolerance and Hydration Status in Newborn Infants

**DOI:** 10.3390/nu7043022

**Published:** 2015-04-22

**Authors:** John Lasekan, Geraldine Baggs, Sonja Acosta, Amy Mackey

**Affiliations:** Abbott Nutrition, Abbott Laboratories, Columbus, OH 43219, USA; E-Mails: geraldine.baggs@abbott.com (G.B.); sonja.acosta@abbott.com (S.A.); amy.mackey@abbott.com (A.M.)

**Keywords:** gastrointestinal tolerance, hydration, term infants, soy infant formulas, fructooligosaccharides, carotenoids

## Abstract

Unlike milk-based infant formulas, soy-based infant formulas containing supplemental fructooligosaccharides (FOS) have not been clinically evaluated. A randomized, double-blind, 28 day parallel feeding trial compared gastrointestinal (GI) tolerance and hydration in healthy term newborn infants fed either a commercialized soy formula (with history of safe use) containing sucrose as 20% of total carbohydrate, no supplemental short-chain FOS (scFOS) and no mixed carotenoids (lutein, lycopene, beta-carotene) as a control (CF, *n* = 62 infants) or one of two experimental soy-based formulas, EF1 (*n* = 64) and EF2 (*n* = 62) containing scFOS (2.5 g/L) and mixed carotenoids. EF1 differed from EF2 by containing sucrose. Results indicated no significant study group differences (*p* > 0.05) in study completion rates (CF = 81, EF1 = 86, & EF2 = 87%), growth, mean rank stool consistency, stool frequency, formula intake, spit-up/vomit, and safety measures (urine specific gravity, USG; hydration status and adverse events). Mean USGs for study groups were normal (<1.03). The EF1 > CF group in percent yellow stools (*p* < 0.01 at age 14 days). In conclusion, the study suggested that term infants fed soy-based formulas supplemented with scFOS and mixed carotenoids, with or without sucrose in the 1st 35 days of infancy demonstrated good tolerance and hydration comparable to the control soy-based formula with history of safe use.

## 1. Introduction

Soy protein-based infant formulas (SF) currently comprise about 13% of total infant formula use in the US [1]. The indication for use of SF includes management of IgE-mediated cow’s milk protein allergy (CMPA), lactose malabsorption or sensitivity, galactosemia, acute diarrhea, and general gastrointestinal discomfort (gas, fussiness and spit-ups); and as a source of nutrition for infants of vegetarian families [1,2]. Despite these SF benefits, studies [3,4,5,6] have demonstrated a firmer stool consistency in infants fed SF compared to those fed milk-based formulas. Dietary ingredients capable of modulating stool consistency may potentially help improve gastrointestinal tolerance and acceptance of SF in infants.

Fructooligosaccharides (FOS) is a non-digestible carbohydrate found in several plant-based foods, including bananas. Oligosaccharides, including FOS and galactooligosaccharides (GOS) are generally considered to be prebiotics because they promote the growth of healthy and beneficial gut *Bifido* and *Lactobacilli* bacteria in the colon [7]. Several clinical studies [8,9,10,11,12,13,14,15] have demonstrated that the supplementation of milk-protein based infant formulas (MF) and infant cereal formulas with FOS and or GOS yielded softer stool consistency compared to non-supplemented formulas. In contrast, there is no reported clinical evaluation of SF supplemented with FOS or GOS. Experts [16] have recommended clinical evaluation of water balance in infants fed formulas with supplemental FOS or GOS as a measure of safety because of the propensity of the supplementation to produce watery stools. Therefore, the supplementation of SFs with FOS and subsequent clinical assessment of GI tolerance and water balance in infants might provide an opportunity to improve soft stool consistency and tolerance of SF. The supplementation of SF with GOS is not advisable so as to avoid the addition of lactose and galactose inherent to GOS ingredients, which are contraindicated in infants with galactosemia or lactose sensitivity.

Most currently available SF contains a minimal amount of sucrose to help enhance palatability, acceptability and compliance by infants in need of the formula. In addition, sucrose masks the beany taste and flavor to help reduce rejection by infants consuming soy-based formula for nutritional or medical purposes. Clinical evidence suggests that sucrose has positive calming and analgesic effects [17,18] on infants. However, it is unclear if sucrose affects GI or stool tolerance in infants.

Dietary carotenoids are lipid soluble compounds found in abundance in fruits and vegetables, and are suggested to be important in immune function, skin and eye health [19,20]. Carotenoids are higher in human milk compared to most infant formulas, and are believed to contribute to the various protection benefits attributed to the breastfed infants [21,22]. Clinical studies have reported a normal growth and tolerance [23,24] in term infants fed milk-based formulas supplemented with carotenoids. One of the studies [23] has demonstrated comparable levels of plasma carotenoids in infants fed milk-based formulas supplemented with carotenoids similar to levels in those fed human milk (HM). However, clinical assessment of a soy-based infant formula with supplemental carotenoids is lacking.

In view of the above, the primary goal of this study was to assess the comparative GI tolerance in healthy term infants fed the two experimental soy-based powdered formulas *versus* a standard commercial control soy-based formula with history of safe use. The experimental formulas were supplemented with scFOS and mixed carotenoids (MC; beta-carotene, lutein and lycopene) while the control formula was not. One of the experimental soy formulas also contains sucrose *versus* none in the other experimental formula.

## 2. Methods

### 2.1. Study Design and Subjects

A randomized, double-blind, multi-center, parallel feeding study was conducted between November 2008 and April 2009. Healthy term infants were enrolled into the study and randomized into one of three study formulas and fed exclusively the assigned study formula from 0–8 days of age to 35 days of age. The primary study variable assessed was mean rank stool consistency (MRSC). Other study variables assessed included weight, length and head circumferences (HC) and their gains; stool consistency, frequency and color; formula intake, spit-up/vomit occurrence, and safety measures (urine specific gravity, USG; hydration status and adverse events).

Subjects were eligible for the study if they were singleton, healthy term infants (gestational age 37–42 weeks) between 0 and 8 days of age, with a birth weight ≥2490 and whose parents have voluntarily confirmed their intention to feed the assigned study formula as the sole source of nutrition for the duration of the study unless instructed otherwise by their health care professionals. Infant subjects were excluded from the study if they had maternal, fetal, or perinatal medical conditions with potential adverse effects on GI tolerance, growth and development, such as diabetes (gestational diabetes was acceptable if the infant’s birth weight was less than the 95th percentile of the Center for Disease Control (CDC) growth standards), intrauterine infections, or suspected substance abuse.

Study centers in the US recruited subjects from the local population at Tampa, FL; Pittsburgh, PA; Lincoln, NE; Plantation, FL; Dalton, GA; Cleveland, OH; Mayfield Heights, OH; Bardstown, KY; St. Petersburg, FL; Dubuque, IA; Cincinnati, OH; Omaha, NE; and Dayton, OH in USA. Subjects were enrolled into the study after parents had voluntarily signed informed consent approved by an institutional review board. The study was approved by the Western Institution Review Board (WIRB, Olympia, WA, USA), a central Institutional Review Board (IRB), for most of the study centers and by individual IRB for the study centers at Lincoln, NE; Little Rock, AR and Cleveland, OH. The first WIRB approval was on 4 September 2008 (#20081120-1106). The study was conducted in accordance with ethical principles that have their origin in the Declaration of Helsinki. The study was also registered with www.clinicaltrials.gov (Registration #NCT00798382).

### 2.2. Study Formulas

The control formula (CF) was Similac^®^ Isomil^®^ Advance^®^ (Abbott Nutrition, Abbott Laboratories, Columbus, OH, USA), a commercially available soy-based powdered infant formula containing sucrose (20% carbohydrate (CHO) as sucrose), but contained no scFOS or mixed carotenoids. The two experimental formulas were (a) a soy-based, powdered infant formula (EF1) containing sucrose (20% CHO as sucrose, 2.5 scFOS g/L), scFOS (NutraFlora FOS; GTC Nutrition; Golden, CO, USA) and mixed carotenoids (MC, beta-carotene, lutein and lycopene); and (b) a soy-based, powdered infant formula (EF2) containing FOS (2.5 scFOS g/L), mixed carotenoids and corn syrup solids (100% CHO as corn syrup solids) but contained no sucrose (Table 1). The MC were crystalline lutein (FloraGlo^®^ Lutein 20%; Kemin Health, Des Moines, IA, USA), synthetic lycopene (LycoVit^®^ Dispersion 20%; BASF, Florham Park, NJ, USA) and synthetic beta-carotene (Lucarotin^®^ 30%; BASF; Florham, NJ, USA). All 3 study formulas contained similar levels of energy (676 Kcal/L or 2826 kJ/L), protein (16.6 g/L), fat (36.9 g/L), minerals and vitamins. Because of scFOS (2.5 g/L; 2 Kcal/g) fortification of EF1 and EF2, and not CF, carbohydrate levels in CF *versus* EF1 and EF2 were 69.7 *versus* 68.45 g/L, respectively. EF1 and EF2 contained similar levels of MC; whereas, CF contained no added MC. All study formulas were iron-fortified infant formulas and met or exceeded the levels of nutrients recommended for term infants by the Committee on Nutrition of the American Academy of Pediatrics [1] and the requirements by the Infant Formula Act of 1980 and its subsequent amendments [25]. The three study formulas were clinically labelled to mask their identity.

**Table 1:** Ingredient Compositional Differences between Study Formulas.

Nutrients	CF	EF1	EF2
Supplemental scFOS, g/L	0	2.5	2.5
Carbohydrate blend, %	20% sucrose	20% sucrose	0% sucrose
	80% corn syrup solids	80% corn syrup solids	100% corn syrup solids
Supplemental Mixed Carotenoids (MC) *	None	Yes	Yes

Abbreviations: CF = Control formula, EF1 = Experimental formula 1, EF2 = Experimental formula 2; * Mixed carotenoids: lutein = 53 mcg/L, lycopene = 81 mcg/L and beta-carotene = 30 mcg/L.

### 2.3. Study Procedures

Enrolled infants were randomly assigned to receive one of the three study formulas starting at 0 to 8 days of age (d1 of study) and fed until day 35 (d35) of age. Parents were trained and instructed to feed the infant the assigned study formula *ad libitum* as the sole source of nutrition and refrain from administration of vitamin or mineral supplements for the duration of the trial. Study assessment were done at Study Visit 1 or Enrollment Visit (at d0–8 of age), Study Visit 2 (at d14 of age), and Study Visit 3/Exit Visit (at d35 of age). Parents were given sufficient amounts of the assigned formula to feed their infant until the next study visit. Parents kept daily records of formula intake (volume and frequency), incidence of spit-up and vomiting associated with feedings, occurrence of fussiness, occurrence of gas, and infant’s stool characteristics (frequency, consistency, color, and odor). Weight, length and HC were measured at d1, d14, and d35 of age, using standard methods [26,27]. Infants were weighed twice in the nude to the nearest 1.0 g on calibrated electronic scales. Infant length was measured twice to the nearest 0.1 cm with the infant in a recumbent position using a pediatric length board. Head circumference was measured in duplicate around the occipital frontal area to the nearest 0.1 cm using an INSER-TAPE (Abbott Nutrition, Abbott Laboratories, Columbus, OH, USA). Urine samples were collected and urine specific gravity (USG) was determined at d14 and d35 of age using the method described by Friedman *et al*. [28]. A physical examination/assessment, which included hydration status, was performed by a study physician or nurse practitioner at d14 and d35 of age. Infant formula satisfaction questionnaire validated and used in published pediatric clinical studies [29,30,31], was completed by parents at d14 and d35 of age to provide ratings of formula acceptability. Interval history interviews were conducted at d14 and d35 of age to identify non-serious adverse events (AEs), serious adverse events (SAEs), consumption of human milk/formula other than study formula and as well as the use of medications, supplements, home remedies or other sources of nutrition.

### 2.4. Statistical Methods

Sample size was estimated assuming standard deviations ranging from 0.6 to 0.8 of the primary variable, mean rank stool consistency (MRSC Score: 1 = watery, 2 = loose/mushy, 3 = soft, 4 = formed, 5 = hard), in similar study populations. Using two-sided multiple comparison tests by Tukey which preserve the family wise error rate for all 3 pair-wise comparisons at 5%, the power is about 80% to detect differences between pairs of means ranging from 0.45 to 0.60 when the total sample size is 117 infants (39 per group). Data were classified and included in the intent-to-treat (ITT) group analyses if collected from subjects who received any of the study formula, and in the evaluable (EV) group analyses if collected from subjects who met study eligibility criteria and also received the assigned study formula. Hypotheses were tested using two-sided, 0.05 level tests. Tests of interactions, when conducted, were two-sided, 0.10 level tests. Holm’s step-down procedure was used to adjust the significance level for multiple comparisons. The Center for Disease Control (CDC) reference data [32] were used to compute standardized z-scores and percentiles for anthropometric variables. Percent data that were not normal were transformed using arcsine of the square root, and/or analyzed non-parametrically. Analysis of variance, Kruskal Wallis test, or repeated measures models were fit to continuous data. Cochran Mantel Haenszel test stratified by site or generalized estimating equations were fit to ordinal data. Adverse events were analyzed using the Fisher’s exact test. All analyses were performed using SAS^®^ Version 9.1 on the Personal Computer.

## 3. Results

Results of demographic and safety measures are presented based on all available randomized subjects, and anthropometric results are presented for both ITT and EV groups. The remaining results are presented based on the EV group analyses because of lesser statistical differences between the study groups in the ITT group analyses.

### 3.1. Disposition, Demographic and Anthropometry Measures among Study Subjects

**Figure 1:**
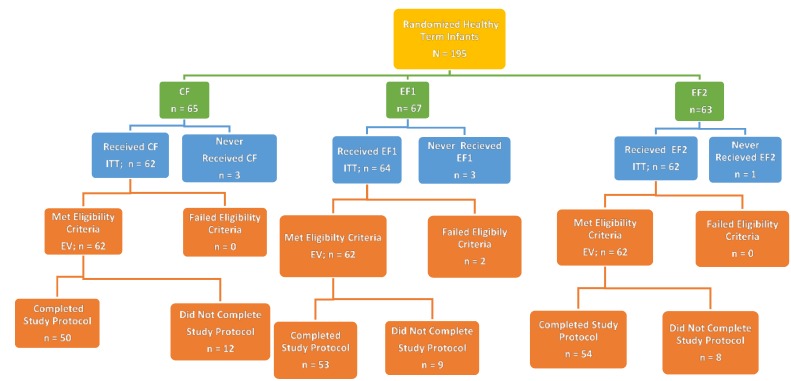
Flow chart of the disposition of study subjects. Abbreviations: CF = Control formula, EF1 = Experimental formula 1, EF2 = Experimental formula 2.

A total of 195 subjects were enrolled into the study; 65, 67 and 63 subjects were in the CF, EF1 and EF2 groups, respectively (Figure 1). Seven subjects did not receive any study product and were excluded from the ITT analyses. The remaining 188 subjects were included in the ITT group. Two subjects on EF1 in the ITT group did not satisfy eligibility criteria by having a birth weight <2490 g or an adverse maternal history that could affect tolerance or growth. They were excluded from the EV group. There were 186 subjects in the EV group. One hundred forty-two subjects were evaluable at d14, and 120 subjects were evaluable at d35. There were no significant differences (*p* > 0.05) between the study formulas in the EV or ITT groups or reasons for the classification. Study completion rates for CF, EF1 and EF2 were 81%, 86% and 87%, respectively. Reasons for not completing the study included the discontinuation of study formula feeding; consumption of non-assigned study or non-study formulas; consumption of fruit juices or supplements, which could affect tolerance; intake of medications that could affect tolerance; missed intake and stool records for more than three days; missed study visits or follow-ups; occurrences of AEs and SAEs; and subjects’ withdrawal from study by parents. However, there were no significant differences between study groups for study completion rates and reasons for non-completion.

Study entrance and demographic data are presented in Table 2. No significant differences (*p* > 0.05) were observed between the three study groups in gender, race, ethnicity, age of subjects at study enrollment, gestational age and birth weight, and birth HC. Birth length in males fed CF was significantly higher than those fed EF2 (*p* = 0.0402), but was not different (*p* > 0.05) with combined males and females. The EF1 group had a higher (*p* = 0.013) rate of vaginal births compared to the CF or EF2 group. Additionally, there were no significant differences (*p* > 0.05) between the three study groups in the type of feeding subjects received before study enrollment at 0–8 days of age. Only one subject was on human milk before study enrollment, and the subject was assigned to the EF2 group. The remaining subjects were on various brands of term infant formulas available in their localities.

Growth as indicated by weight, length, HC and their respective gains were not significantly different among study groups (Table 3). Mean weight percentiles ranged between 24 and 47, length between 38 and 60, and head circumference between 28 and 40 on the 2000 CDC Growth Charts.

**Table 2:** Demographic and entrance data for randomized study subjects.

Variables	Treatment Groups		
	CF	EF1	EF2
Gender			
Male/Female, *n* (%)	33/32 (51/49)	32/35 (48/52)	30/33 (48/52)
Race, *n* (%)			
White	42 (65)	45 (67)	37 (59)
Black	15 (23)	12 (18)	23 (37)
Asian	0 (0)	1 (1)	0 (0)
Pacific Islander	0 (0)	1 (1)	0 (0)
White/Hispanic	0 (0)	1 (1)	0 (0)
White/Black	7 (11)	6 (9)	3 (5)
White/Asian	0 (0)	1 (1)	0 (0)
Black/Asian	1 (2)	0 (0)	0 (0)
Gestational Age, weeks *	39.1 ± 0.1 (65)	38.9 ± 0.1 (67)	38.9 ± 0.1 (62)
Mode of Delivery **			
Vaginal/C-Section, *n* (%)	42/20 (68/32)	54/10 (84/16)	40/22 (65/35)
Age at Enrollment, days	5.3 ± 0.2 (65)	4.7 ± 0.2 (67)	5.3 ± 0.2 (63)
Birth Weight, g	3382 ± 57 (65)	3345 ± 52 (67)	3211 ± 51 (63)
Birth Length, cm ^‡^	50.3 ± 0.3 (65)	50.5 ± 0.3 (67)	49.8 ± 0.2 (63)
Birth Head Circumference, cm	33.8 ± 0.4 (35)	34.0 ± 0.3 (48)	33.9 ± 0.2 (39)

Abbreviations: CF = Control formula, EF1 = Experimental formula 1, EF2 = Experimental formula 2; * Values are mean ± SEM (*n*). No significant differences (*p* > 0.05); ** Mode of delivery was significantly higher for EF1 *versus* CF or EF2 (*p* = 0.013); ^‡^ Males: CF > EF2 (*p* = 0.0402; CF = 50.9 ± 0.4 > EF2 = 49.6 ± 0.4).

**Table 3:** Anthropometric values for study subjects *.

Study Variables	Treatment Groups		
	CF	EF1	EF2
**Evaluable (EV) Group**			
Weight			
Day 1, g	3297 ± 58 (62)	3318 ± 49 (62)	3150 ± 48 (62)
Day 1, percentile	32.4 ± 3.0 (62)	33.4 ± 2.8 (62)	24.3 ± 2.4 (62)
Day 35, g	4307 ± 73 (37)	4302 ± 72 (39)	4190 ± 70 (44)
Day 35, percentile	43.3 ± 3.4 (37)	44.7 ± 3.6 (39)	36.2 ± 3.5 (44)
Weight gain, g/day			
Days 1–35	35.8 ± 1.9 (37)	34.0 ± 1.7 (39)	35.3 ± 1.6 (44)
Length gain, cm/day			
Days 1–35	0.14 ± 0.01 (37)	0.14 ± 0.01 (39)	0.14 ± 0.01 (44)
Head circumference gain, cm/day			
Days 1–35	0.09 ± 0.00 (37)	0.10 ± 0.01 (39)	0.09 ± 0.00 (44)
**Intent-to-Treat (ITT) Group**			
Weight			
Day 1, g	3297 ± 58 (62)	3311 ± 50 (64)	3150 ± 48 (62)
Day 1, percentile	32.4 ± 3.0 (62)	33.2 ± 2.8 (64)	24.3 ± 2.4 (62)
Day 35, g	4339 ± 68 (50)	4373 ± 66 (54)	4213 ± 60 (54)
Day 35, percentile	44.1 ± 3.3 (50)	46.6 ± 3.3 (54)	37.5 ± 3.1 (54)
Weight gain, g/day			
Days 1–35	35.1 ± 1.6 (50)	34.7 ± 1.4 (54)	35.6 ± 1.4 (54)
Length gain, cm/day			
Days 1–35	0.13 ± 0.01 (49)	0.15 ± 0.01 (54)	0.14 ± 0.01 (54)
Head circumference gain, cm/day			
Days 1–35	0.09 ± 0.00 (50)	0.10 ± 0.01 (54)	0.10 ± 0.00 (54)

Abbreviations: CF = Control formula, EF1 = Experimental formula 1, EF2 = Experimental formula 2; * Values are mean ± SEM (*n*). No significant differences (*p* > 0.05).

### 3.2. Stool and Gastrointestinal Tolerance and Formula Acceptability

**Figure 2:**
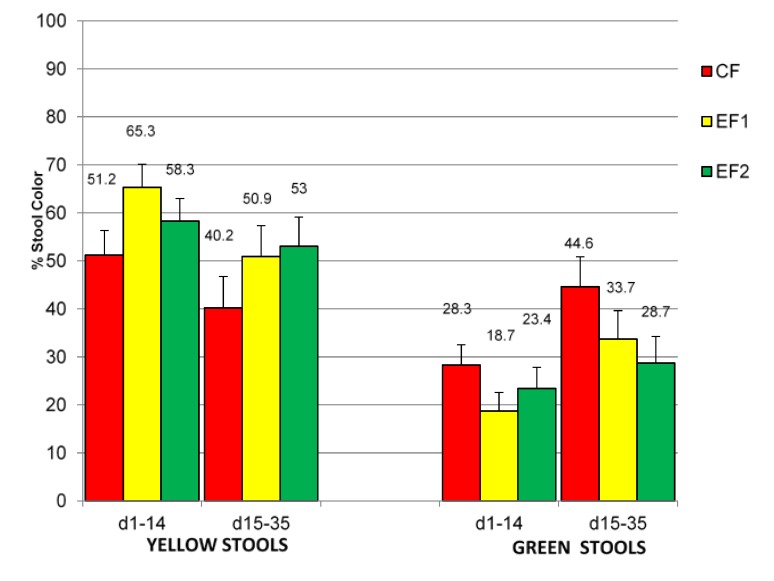
Percentages of yellow or green stools produced by evaluable (EV) subjects. Abbreviations: CF = Control formula, EF1 = Experimental formula 1, EF2 = Experimental formula 2. * Percentages of yellow stools for EF1 > CF (*p* = 0.0314) at d1–14.

Mean rank stool consistency (MRSC) was not significantly different (*p* > 0.05) among the 3 study formula groups from d1 to 35 days of age (Table 4). MRSC did not change with the progression of feeding during the course of the study. There were also no significant differences in the average number of stools per day and predominant stool consistency, color, odor and gassiness observed among the study groups. The percentage of hard stools was significantly higher (*p* = 0.0207) in EF1 *versus* EF2 group at d1–14, but not at d15–35 (Table 4). However, no significant differences were noted between study groups in percentages of watery stools, loose/mushy stools, soft stools, and formed stools. The predominant stool consistency for CF and EF2 was loose/mushy and that for EF1 was soft, but the difference was not statistically different (*p* > 0.05). The percentages of stool color that were yellow were significantly higher (*p* = 0.0314) in EF1 *versus* CF group at d1-14, but not at d15–35 (Figure 2). However, the trend for a higher percentage of yellow stools noted in EF1 group was still apparent at d15–35 despite the absence of statistical significance. Conversely, percentages of green stool were numerically higher in CF *versus* EF1 or EF2 group but not significantly different (*p* > 0.05). The percentages of brown stools and black stools were not different in the study groups.

There were also no significant differences observed among the study groups in formula intake volume and percent of formula feedings with spit-up and/or vomit within 1 hour of feeding (Table 5). The EF2 group had significantly higher average numbers of feedings per day compared to the EF1 group at d1–14 but not at d15–35. Formula intake by the 3 study groups increased from d1–14 to d15–35; whereas, numbers of feeding per day and the percent of formula feedings with spit-up and/or vomit decreased in the 3 study groups from d1–14 to d15–35. Most parental responses to the formula satisfaction questionnaire were not significantly different (*p* > 0.05). Parental responses to the questionnaire on how well study powder mix with water, yielded a significantly higher (*p* < 0.05) positive response for CF *versus* EF1 group. Furthermore, responses to the questionnaire on baby gassiness indicated that the EF1 group was significantly less gassy compared to EF2 at d14.

**Table 4:** Stool characteristics in study evaluable (EV) subjects *.

Study Variables	Treatment Groups		
	CF	EF1	EF2
Mean Rank Stool Consistency Score ** (Primary Study Variable)			
Days 1–14	2.5 ± 0.1 (51)	2.6 ± 0.1 (57)	2.5 ± 0.1 (57)
Days 15–35	2.6 ± 0.1 (40)	2.7 ± 0.1 (41)	2.5 ± 0.1 (46)
Stool Frequency, # stools/day			
Days 1–14	2.9 ± 0.3 (48)	3.3 ± 0.3 (55)	3.1 ± 0.3 (56)
Days 15–35	2.1 ± 0.2 (40)	2.7 ± 0.3 (41)	2.7 ± 0.3 (46)
Percent Watery Stools, %			
Days 1–14	11.3 ± 2.6 (51)	16.3 ± 3.4 (57)	12.7 ± 2.8 (57)
Days 15–35	8.9 ± 2.0 (40)	11.4 ± 3.4 (41)	13.3 ± 3.4 (46)
Percent Loose/Mushy Stools, %			
Days 1–14	47.7 ± 4.7 (51)	32.9 ± 3.9 (57)	39.5 ± 3.7 (57)
Days 15–35	40.4 ± 5.5 (40)	31.7 ± 4.5 (41)	41.0 ± 4.7 (46)
Percent Soft Stools, %			
Days 1–14	26.7 ± 3.7 (51)	34.8 ± 3.9 (57)	37.5 ± 4.1 (57)
Days 15–35	35.3 ± 5.1 (40)	40.1 ± 4.3 (41)	30.3 ± 4.6 (46)
Percent Formed Stools, %			
Days 1–14	10.8 ± 3.0 (51)	10.4 ± 1.9 (57)	9.0 ± 2.3 (57)
Days 15–35	11.4 ± 2.8 (40)	15.5 ± 2.9 (41)	12.6 ± 2.9 (46)
Percent Hard Stools, % ^‡^			
Days 1–14	3.5 ± 1.7 (51)	5.6 ± 2.5 (57)	1.3 ± 0.9 (57)
Days 15–35	4.0 ± 2.2 (40)	1.4 ± 0.6 (41)	2.8 ± 1.4 (46)

Abbreviations: CF = Control formula, EF1 = Experimental formula 1, EF2 = Experimental formula 2; * Values are mean ± SEM (*n*); ** Mean Rank Stool Consistency Score: 1 = watery, 2 = loose/mushy, 3 = soft, 4 = formed, 5 = hard; ^‡^ Percent of Hard Stools at d1-14; EF1 > EF2 (*p* = 0.0207).

**Table 5:** Gastrointestinal tolerance of study formulas in evaluable (EV) subjects *.

Study Variables	Treatment Groups		
	CF	EF1	EF2
Average Numbers of Feedings, #/day **			
Days 1–14	7.6 ± 0.2 (47)	7.1 ± 0.2 (56)	7.9 ± 0.2 (56)
Days 15–35	7.1 ± 0.2 (40)	7.1 ± 0.2 (40)	7.5 ± 0.3 (47)
Average Formula Intake, mL/day			
Days 1–14	555 ± 17 (47)	559 ± 20 (56)	570 ± 20 (56)
Days 15–35	673 ± 22 (40)	739 ± 26 (40)	726 ± 35 (47)
Spit-up/Vomit, % of feedings			
Days 1–14	22.4 ± 4.0 (52)	23.5 ± 3.8 (58)	23.2 ± 3.9 (59)
Days 15–35	17.4 ± 4.1 (40)	17.8 ± 4.1 (40)	17.5 ± 3.6 (47)

Abbreviations: CF = Control formula, EF1 = Experimental formula 1, EF2 = Experimental formula 2; * Values are mean ± SEM (*n*); ** Average Numbers of Feedings at d1–14; EF2 > EF1 (*p* = 0.0215).

### 3.3. Safety Measures

Safety measures including SAEs, AEs, hydration status, and urine specific gravity were not significantly different between the study formula groups (Table 6). A total of 6 SAEs were reported in the study, 2 in each study group; and were rated as “not related” or “probably not related” to study formulas by study investigators. Only one of the 6 subjects with SAE (in the CF group) failed to complete the study. No deaths were reported in the study. The number of parental reports of loose/watery stools in the CF, EF1, and EF2 groups were 4, 7 and 2, respectively. However, they were not significantly (*p* > 0.05) different, and the hydration status and urine specific gravity for these subjects were normal (normal value is <1.030 [28]). Oral candidiasis infection rate was significantly (*p* = 0.0081) less in the EF1 group compared with CF (CF = 11, EF1 = 2, and EF2 = 6).

**Table 6:** Urine specific gravity and serious adverse events for study subjects *.

	Treatment Groups		
	CF	EF1	EF2
**Urine Specific Gravity**			
Day 14			
Mean ± SEM (*n*)	1.0041 ± 0.0005 (40)	1.0038 ± 0.0004 (42)	1.0044 ± 0.0009 (47)
Abnormal USG (≤1.030 **) *n* (%)	None	None	1 (2)
Day 35			
Mean ± SEM (*n*)	1.0043 ± 0.0004 (35)	1.0034 ± 0.0003 (38)	1.0039 ± 0.0004 (43)
Abnormal USG ≤ 1.030 ** *n* (%)	None	None	None
**Serious Adverse Events (SAEs)**			
Total Number of Subjects	(*N* = 62)	(*N* = 64)	(*N* = 62)
Subjects with SAEs, *n* (%)	2 (3.2)	2 (3.1)	2 (3.2)
SAE Complaint/Diagnosis	Respiratory syncytial virus Bronchiolitis Pyrexia	Streptococcal sepsis Urinary tract infection	Meningitis enterovirus Vomiting

Abbreviations: CF = Control formula, EF1 = Experimental formula 1, EF2 = Experimental formula 2; * No significant differences (*p* > 0.05); ** Friedman *et al.* [28].

## 4. Discussion

The primary goal of the study was to assess GI tolerance to soy based infant formulas with supplemental scFOS using measures of stool tolerance in infants. To our knowledge, this study is the first reported clinical evaluation of a soy protein-based infant formula, supplemented with FOS. Clinical studies in infants and young children have suggested that foods with supplemental FOS modulate stool consistency. Moore *et al*. [8] demonstrated softer stools, increased stool frequencies; fewer complaints of hard stools or constipation, and good GI tolerance in infants consuming FOS supplemented baby cereals. Euler *et al*. [9] evaluated the effect of 1.5 and 3.0 g/L FOS supplementation of milk-based infant formulas in a randomized crossover study in normal infants for one week. The 3.0 g/L FOS supplementation resulted in more frequent and significantly softer stools compared to the control. Both levels of FOS supplementation were reported by the authors to be safe and well tolerated. Paineau *et al*. [14] compared milk-based formulas supplemented with 4.0 g/L scFOS *versus* placebo supplemented with 4.0 g/L maltodextrins in healthy infants. They reported significantly higher change in bifido bacteria population in stools of infants fed the scFOS compared to the placebo group. Other investigators have conducted studies on milk-based infant formulas with combination of FOS and GOS in both term [10] and preterm infants [11]. The addition of GOS to milk-based infant formulas, fed alone [12] or in combination with oligofructose at a 9:1 ratio [13,15], resulted in normal growth, stool softening, and beneficial effects on health and development of newborn infants.

In our current study, we did not see any effect of the supplemental scFOS at 2.5 g/L on stool softening as indicated by the absence of a significant difference in the MRSC among the 3 study formulas. It is quite possible that a higher level of scFOS supplementation maybe needed for the soy-based formulas to produce a softer stool consistency compared to the level required for the milk-based formulas. It is well known that soy-based formulas tend to produce firmer stools compared to milk-based formulas or HM [3,4,5,6]. This could be partly due to the higher levels of inherent fiber and raffinose and starchyose in soy protein isolate components of soy formulas and the need for a higher calcium fortification rate in soy formulas relative to milk-based formulas. A modest level of scFOS supplementation was used in this pilot study to avoid a possible excessive watery stools and water balance issues when a higher level of oligosaccharides is used in infant formulas as recommended against by Experts [16].

The results of the current study indicated that the two experimental formulas were generally well tolerated and were favorably comparable to the established control formula. This is supported by the absence of significant differences (*p* > 0.05) in many of the GI tolerance measures evaluated in this study. Nonetheless, there were some measures that slightly favored the experimental formulas *versus* the control formula. The EF1 and EF2 groups produced significantly more yellowish stools *versus* the CF group throughout the study; although statistically significant differences were noted only at d1–14 and not at d15–35 periods. The study period differences could be possibly due to adaptation with the progression in feeding and age of the infant subjects and the diminishing subjects’ sample size towards the end of study. The EV group had 142 subjects at d14, which was reduced to 120 subjects at d35. Notwithstanding, the yellowish stool trend was present and consistent throughout the study periods. The production of yellowish stools may be desirable for infant formula-fed infants, because stools produced by human milk-fed infants are predominantly yellowish compared to those produced by formula-fed infants [4,33]. Other studies which evaluated infant formulas supplemented with a combination of GOS and FOS [15], and rice starch [30] have similarly noted an increase in yellowish stools when compared to formulas that were not supplemented. The rationale for the production of yellowish stools is unclear. However, it is likely related to the prebiotic effects of oligossacharides on bifidogenic bacteria in the GI tract. Study completion rates were slightly better for EF1 and EF2 groups compared to the CF group despite a lack of statistically significant differences (CF, EF1 and EF2 were 81%, 86% and 87%, respectively).

There were no statistically significant differences between the study groups in the safety measures (AEs, SAEs, hydration status and physical examinations) assessed in this study. Despite the observed numerically higher (but no statistically significant differences) parental report of the occurrence of diarrhea in the EF1 group relative to the CF or EF2 group, the hydration status as denoted by the USG for the subjects with diarrhea were normal. The observation of a lower oral candidiasis infection in the EF1 group *versus* EF2 and CF groups is interesting but its clinical relevance is unclear. It is unknown if this observed difference in infection rate is related to the higher vaginal births noted with subjects in the EF1 *versus* EF2 and CF groups. Nonetheless, this finding is consistent with studies [34,35] which have reported a higher risk of infection in infants who were born by caesarean compared to those born by vaginal routes.

Human milk and some infant formulas have introduced carotenoids into the infant diets. A recent study [23] of milk-based formulas supplemented with similar levels of mixed carotenoid (MC) in our current study demonstrated comparable blood levels of lutein, lycopene and beta-carotene as in human milk-fed infants. The milk-based formulas supplemented with MC were well tolerated and supported normal growth. To our knowledge, our current study is the first study to report the supplementation of a soy-based infant formula with MC. The supplementation of EF1 and EF2 formulas with MC did not negatively impact GI tolerance.

An additional secondary interest of the current study was to evaluate the impact of sucrose inclusion in soy-based formula on formula tolerance in normal infants. Clinical evidence suggests that sucrose has positive calming and analgesic effects [17,18] on infants, especially infants with tolerance issues; however, it is unclear if sucrose affects stool patterns in infants. For the most part, in this study, tolerance responses were similar between the two experimental formulas, which only differ by having (EF1) or not having sucrose (EF2). The few differences observed between EF1 and EF2 included a significantly higher percentage of hard stools with EF1 *versus* EF2 at d14 but not at d35 of age, which suggests a possible transient effect; and a significantly more parental response favoring an “intent to continue to use study formula” for the EF2 *versus* EF1 group. In contrast, the parental questionnaire response indicated that the EF1 group was significantly less gassy compared to EF2 at only 14 days of age and not at 35 days of age. EF1 also had a slightly higher (non-statistically significant) percentage of yellowish stool color compared to EF2 group. Nonetheless, both formulas were well tolerated, using the CF formula as the standard comparator. The true impact of sucrose on GI tolerance in infants can best be evaluated in infants experiencing GI intolerance or colic symptoms. In our current study, we only enrolled normal infant subjects who were not having tolerance issues.

The strength of this current study includes being the first clinical study to assess the GI tolerance and short-term safety impact of soy protein-based infant formula supplemented with FOS in healthy term infants, and also the first clinical study to provide documented clinical GI tolerance feeding experience for soy protein-based infant formulas supplemented with MC. Among the weakness of the study are the absence of an assessment of varying levels of supplemental scFOS, which might potentially have a robust effect on GI tolerance; the absence of human milk and or milk protein-based formula group(s) to serve as a reference in the current study; the non-assessment of stool chemistry and probiotic colonization effects of the supplemental scFOS in the study; the shorter feeding duration of feeding in the study; and that the study did not address the efficacy of these types of formulas in infants experiencing tolerance issues. However, the intent of the current study was a pilot study in scope. These identified weaknesses are likely to be addressed by future studies.

## 5. Conclusions

In conclusion, this study is unique by being the first clinical study to evaluate soy protein-based infant formulas with supplemental scFOS prebiotics and supplemental mixed carotenoids. There were no significant differences in MRSC, GI tolerance, growth, and safety measures (including hydration) between the supplemented formulas *versus* the control commercial formula, which has a long history of safe use. Consequently, this study demonstrated that the addition of ScFOS at 2.5 g/L and mixed carotenoids to soy protein-based infant formulas, with or without sucrose are suitable and well tolerated by healthy term infants when fed in the first 35 days of infancy.
